# Having mental health problems but not sickness absent: factors of importance among privately employed: Jurgita Narusyte

**DOI:** 10.1093/eurpub/ckac130.213

**Published:** 2022-10-25

**Authors:** J Narusyte, I Alaie, A Ropponen, P Svedberg

**Affiliations:** 1 Division of Insurance, Karolinska Institutet, Stockholm, Sweden; 2 Centre for Epidemiology and Community Medicine, Stockholm, Sweden; 3 Finnish Institute of Occupational Health, Helsinki, Finland

## Abstract

**Background:**

Knowledge is scarce on individuals who are experiencing mental health problems but who have low or no sickness absence (SA). The aim of this study was to identify individual-level characteristics, including sociodemographic factors, morbidity, and lifestyle, among privately employed individuals with previous depression/anxiety but no SA during follow-up.

**Methods:**

This prospective cohort study included 750 twin individuals born in Sweden in 1959-1986, employed in the private sector and with a history of depression/anxiety. Depending on the birth year, the twins were invited to participate in two different health-screening surveys in 2005, when study participants were aged 19-20 or 20-30, respectively. Survey data were used to evaluate depression and anxiety, self-rated health, stressful life events, emotional neglect, level of physical activity, and alcohol use. Study participants were prospectively followed regarding SA occurrence between 2006 and 2018. Data on SA, sociodemographic factors, outpatient healthcare use, and use of prescribed antidepressants were obtained from the Swedish national registries. Descriptive statistics will be reported with further analyses for the presentation.

**Results:**

Preliminary results showed that despite previous depression or anxiety, 35% of women and 52% of men were not on SA during the follow-up period. Those who had no SA during follow-up were more likely to have higher education >12 years (49%), experienced fewer stressful life events (43%) and emotional neglect (56%), had better self-rated health (95%), along with a lower use of antidepressants (11%) and outpatient healthcare (88%), as compared with those on SA (33%, 65%, 66%, 90%, 17%, and 98%, respectively).

**Conclusions:**

Higher education, being male, fewer life adversities, good self-rated health and low use of antidepressants and outpatient healthcare were individual-level factors of importance for those with previous depression or anxiety and no incident SA during follow-up.

**Key messages:**

- Individuals with previous depression/anxiety, but not sickness absent, were more likely to have higher education, be male, and have lower morbidity, as compared to individuals on sickness absence.
- Identification of factors that prevent sickness absence among those with mental health problems may potentially be used to improve sickness absence prevention strategies.

